# Follicular Fluid: A Powerful Tool for the Understanding and Diagnosis of Polycystic Ovary Syndrome

**DOI:** 10.3390/biomedicines10061254

**Published:** 2022-05-27

**Authors:** Ana Teresa Brinca, Ana Cristina Ramalhinho, Ângela Sousa, António Hélio Oliani, Luiza Breitenfeld, Luís A. Passarinha, Eugenia Gallardo

**Affiliations:** 1 Health Sciences Research Centre, Faculty of Health Sciences, University of Beira Interior, 6200-506 Covilhã, Portugal; ana.brinca@ubi.pt (A.T.B.); angela@fcsaude.ubi.pt (Â.S.); luiza@fcsaude.ubi.pt (L.B.); 2 Assisted Reproduction Laboratory of Academic Hospital of Cova da Beira, 6200-251 Covilhã, Portugal; aholiani@gmail.com; 3 C4-Cloud Computing Competence Centre, University of Beira Interior, 6201-001 Covilhã, Portugal; 4 São José do Rio Preto School of Medicine, Gynaecology and Obstetrics, São José do Rio Preto 15090-000, Brazil; 5 UCIBIO–Applied Molecular Biosciences Unit, Departament of Chemistry, NOVA School of Science and Technology, Universidade NOVA de Lisboa, 2829-516 Caparica, Portugal; 6 Associate Laboratory i4HB-Institute for Health and Bioeconomy, NOVA School of Science and Technology, Universidade NOVA de Lisboa, 2819-516 Caparica, Portugal; 7 Laboratório de Fármaco-Toxicologia, UBIMedical, University of Beira Interior, 6200-284 Covilhã, Portugal

**Keywords:** polycystic ovary syndrome (PCOS), human follicular fluid, metabolomics, reproduction

## Abstract

Polycystic ovary syndrome (PCOS) represents one of the leading causes of anovulatory infertility and affects 5% to 20% of women worldwide. Until today, both the subsequent etiology and pathophysiology of PCOS remain unclear, and patients with PCOS that undergo assisted reproductive techniques (ART) might present a poor to exaggerated response, low oocyte quality, ovarian hyperstimulation syndrome, as well as changes in the follicular fluid metabolites pattern. These abnormalities originate a decrease of Metaphase II (MII) oocytes and decreased rates for fertilization, cleavage, implantation, blastocyst conversion, poor egg to follicle ratio, and increased miscarriages. Focus on obtaining high-quality embryos has been taken into more consideration over the years. Nowadays, the use of metabolomic analysis in the quantification of proteins and peptides in biological matrices might predict, with more accuracy, the success in assisted reproductive technology. In this article, we review the use of human follicular fluid as the matrix in metabolomic analysis for diagnostic and ART predictor of success for PCOS patients.

## 1. Introduction

Polycystic ovary syndrome (PCOS) is one of the prevailing gynecological and endocrine and metabolic disorders among women of reproductive age, representing one of the leading causes of anovulatory infertility. According to the criteria in practice, this disease affects 5% to 20% of women worldwide [1,2,3,4].

According to the Rotterdam Consensus on Diagnostic Criteria for PCOS, this is a multifaceted pathology that encompasses many clinical manifestations. It is necessary to present two out of three of these characteristics in order to be diagnosed with PCOS: oligoanovulation [5]; clinical and/or biochemical hyperandrogenism (HA) [6]; polycystic ovarian morphology that does not encompass other abnormalities, such as Cushing’s syndrome, congenital adrenal hyperplasia, and androgen-secreting tumors [7].

To be considered a woman with polycystic ovary morphology, it is necessary to present at least one ovary with “12 or more follicles, measuring 2–9 mm in diameter, and/or increased ovarian volume, namely above 10 mL” [8,9,10,11,12]. This definition, however, does not apply to women who use the oral contraceptive pill because its use alters ovarian morphology [8,13]. A scan should be repeated in the next cycle if a corpus luteum or dominant follicle are discovered. Any evidence of ovarian asymmetry or an abnormal cyst requires a more in-depth inquiry, as well as asymptomatic polycystic ovarian morphology women, since they do not present ovulatory disorder or hyperandrogenism [8,14,15,16].

Metabolic abnormalities are also common, even if they are not considered to classify a PCOS woman. Three out of the following five qualify for metabolic syndrome: blood pressure [8,17,18]; abdominal obesity (waist circumference) [8,17,19]; high levels of triglycerides (TG) [8,17]; fasting and two-hours glucose from oral glucose tolerance test; low levels of HDL-C. These might further generate an increased risk of type 2 diabetes [17,20,21], insulin-resistance (IR) [17,22,23], and cardiovascular diseases [17,24,25,26,27,28], and they might disturb the body mass index (BMI) [8,17].

Because of the variety of clinical and biochemical manifestations of PCOS, two of the hallmarks of these women, which heavily influence the disease phonotypy, are overweight and obesity, with only 30–50% of PCOS patients exhibiting an average weight. Such conditions may result in IR and metabolic syndrome [2]. Glucose intolerance and an oral glucose tolerance test should be used to screen obese women with PCOS for metabolic syndrome. IR is described as the decrease in the use of glucose mediated by insulin. This metabolic abnormality occurs in more than 50% of PCOS cases and leads to reproductive complications. Improving the lifestyle and recovering with pharmacological intervention can help mitigate further irregularities [8,29]. Insulin can keep lipid metabolism in check by blocking the release of free fatty acids from adipose tissue, and in patients with IR, inhibition of the lipid oxidation rate is weakened, resulting in an increase in the concentration of free fatty acids in FF [4,30,31]. Some criteria for defining a metabolic syndrome were developed. These include components associated with the IR syndrome, such as centripetal obesity, hypertension, fasting hyperglycemia, and dyslipidemia [17,20,21,32]. It was proposed to add an oral glucose tolerance test (OGTT) to the fasting blood tests. The 2-h glucose level after a 75-g oral glucose challenge for glucose intolerance could be evaluated in this manner. Within obese PCOS women, impaired glucose tolerance and type 2 diabetes are two common features, both being diagnosed by OGTT [8,21]. IR, common in PCOS patients, increases the risk of metabolic syndrome and cardiovascular morbidity, and it generates higher glucose concentrations [3,33]. PCOS women that present obesity, a family history of type 2 diabetes, IR, or beta-cell dysfunction have high probabilities of developing diabetes [20,21,22,34,35]. Apart from these metabolic abnormalities, it is also suggested that these woman present an increased risk of having strokes and cardiovascular diseases [8,24,36]. IR is linked to coronary heart disease, and PCOS patients might also present dyslipidemia and abnormal vascular function [37,38,39,40,41,42,43]. Chronic anovulation with unopposed estrogen exposure to the endometrium has also been linked to an increased risk of endometrial cancer [8,44]. Other aspects may be considered as additional risk factors, such as a family history of diabetes [8]. Clinical studies have revealed that PCOS patients with weight and BMI reduction were often associated with menstrual bleedings, the return of ovulation, and the normalization of other metabolic parameters [4,45].

One of the key features of PCOS is the presence of clinical and/or biochemical androgen excess, aside from other diseases [8,46]. Some clinical features of hyperandrogenism encompass the presence of hirsutism, acne, and androgenic alopecia when coupled with oligoovulation [8,46,47,48]. In addition, some studies pointed out that the circulating androgen levels, corresponding to the biochemical fraction, might represent an inherited marker for androgen excess, even though this aspect may not be clear in all cases [49,50,51,52,53,54]. However, the quantification of the different androgens is challenging, since most of the commercially available kits are inaccurate [8,55,56,57], and not all chromatographic methods allow the precise detection of these hormones [58,59,60]. Additionally, multiple androgens that are characterized through these methods cannot be considered in the final evaluation [8,61]. Due to the wide variety of the population, the values of the hormones may differ more than expected, and, therefore, control limits have not yet been set [8,28,62]. Another pertinent point is that androgen suppression is extremely difficult to reverse, even after hormonal treatment has been discontinued [8]. The evaluation of free androgens and their index are sensitive markets for assessing hyperandrogenemia. The first, however, may not be the most accurate in determining androgen excess [8,57,63,64,65].

Due to an increase in the amplitude and frequency of LH pulses, LH concentrations and their relationship to FSH levels are immensely elevated in PCOS women [8,66,67,68]. These levels may be influenced by the timing of ovulation, BMI (being lower in PCOS women with a higher BMI), and the analytical method used. The effects of LH on human reproduction are highly debated, with some studies suggesting that high levels of this hormone may reduce oocyte maturation and fertilization, resulting in higher miscarriage rates [8,67,69,70]. Others concluded that abnormal LH concentrations did not affect oocyte and embryo quality, implantation, fertilization, or pregnancy outcomes [8,71,72]. Many studies show GnRH being used to reduce endogenous LH. However, there was some disagreement because some studies suggested that this practice reduced miscarriage rates [8,73], while others questioned such a therapeutic effect [8,74,75]. Nonetheless, LH can be used as a secondary parameter, particularly in women who are not overweight [8]. Until today, both the subsequent etiology and pathophysiology of PCOS remain unclear. To reduce the psychological and social pressures that come with this pathology, several studies were conducted to facilitate the diagnosis and treatments [1,76]. Patients with PCOS that undergo assisted reproductive techniques (ART) might present a poor to exaggerated response, low oocyte quality, ovarian hyperstimulation syndrome, as well as changes in the FF metabolites pattern. These abnormalities originate a decrease of MII oocytes and decreased rates for fertilization, cleavage, implantation, blastocyst conversion, poor egg to follicle ratio, and increased miscarriages [1,2]. Over the years, the focus of research shifted from embryo to oocyte quality to optimize IVF outcomes and to improve pregnancy rates. However, since PCOS is considered a heterogenous disease, obtaining high-quality embryos is taken into more consideration [1,33], where every patient should undergo custom tests to receive the most accurate guidance.

## 2. Follicular Fluid (FF)

Follicular fluid (FF) serves as a complex microenvironment for germ cell–somatic cell communication. It encompasses a variety of metabolites and enables different reactions to take place that are crucial to oocyte growth [2]. It is derived from the diffusion of serum, transudate of plasma, and metabolites synthesized in the follicle wall that will later be altered by granulosa cells (GCs) and theca cells. In addition, compounds that derive from local follicular metabolic processes and the biological activities of ovarian cells are also present. This biological matrix is the only one directly associated with the oocyte since it is where its growth and differentiation occurs in vivo. It contains a variety of bioactive molecules that change in quantity and quality during follicle development, as well as specific changes in the follicular microenvironment that lead to follicle and oocyte maturation and development. The biosynthesis and transport of these metabolites are crucial to multiple metabolic reactions; they regulate meiosis, are involved in the synthesis of steroid hormones and glycoproteins by the dominant follicle, and promote follicle and oocyte maturation and development, fertilization, and implantation [77,78,79]. The follicle wall acts as a highly rough molecular sieve that allows passage to small metabolites while restringing the access to molecules over 100 kDa. There is bidirectional signal regulation and metabolite transport between GCs and oocytes, such as steroid hormone biosynthesis, oocyte gene transcription, and protein synthesis regulation, showing a deep connection between the oocyte and GCs [78].

Some research also points to the role of oxidative stress in follicular fluid as a causative factor of female infertility. FF contains reactive oxygen species (ROS) and antioxidant enzymes, with ROS being physiologically produced during the ovulatory process. Under physiological conditions, antioxidant defense systems prevent ROS production and scavenge existing free radicals. It is proposed that ROS significantly speed up ovarian aging. Proteins are among the most affected metabolites by ROX, and carbonyl groups (aldehydes and ketones) are produced on protein side chains when oxidized. Carbonylated proteins are formed early in oxidative stress conditions and are relatively stable. They induce stable modifications at the protein level (identified as oxidative damage to the proteins), leading to the proteolytic breakdown of the peptide bond, crosslinking and/or modifications of amino acids such as carbonylation, or the formation of disulfide bridges intra and intermolecularly. These changes may alter protein function and antigenicity, potentially inducing immunological processes associated with inflammation and autoimmune damage. This faulty immune system activation regulation, and the unbalanced production of ROS, may result in oxidative damage. Many pathological conditions affecting female fertility may derive from the synergistic action of oxidative stress and immunity [77,80,81,82,83,84,85,86,87,88,89,90,91,92,93,94,95,96,97,98,99,100,101,102,103,104,105].

A more detailed understanding of FF and its metabolic profile is crucial for further analysis of several pathologies, such as PCOS, endometriosis, and early ovarian failure. FF has become an essential source of information since it is a non-invasive matrix that gathers biological insights about fertility, reflecting the alterations of the patient’s microenvironment. Recently, the molecular and biomolecular signature of FF have aroused many interests, leading to several studies that aimed to identify new targets that allow for evaluation of the development of the oocyte. Consequently, an exhaustive characterization and comprehension of FF may then help the recognition of metabolites that could potentially disturb normal female function and promote infertility [78,79,106].

## 3. Metabolomics in PCOS

Conventional approaches used to determine and analyze biomarkers related to oocyte development, and to predict its quality and viability, tend to be ineffective, imprecise, and present some analytical limitations. Thus, the quantification of proteins and peptides in biological matrices through metabolomic analysis might predict, with more accuracy, the success of an assisted reproductive procedure [78]. Metabolomics is a high-throughput method for detecting several metabolic contents in diverse biological samples. Untargeted metabolomics, which focuses on the dynamic changes of all small molecules in response to an organismal disturbance, can provide deep insights into etiopathogenesis and the recognition of different biomarkers for a variety of diseases [2]. In general, metabolomics is a recent field of ‘omics’ technology that arises from genomics, transcriptomics, and proteomics. It is a growing field that has been subject to much consideration regarding integrative analysis. Metabolites are described as low-molecular-weight molecules (<1500 Daltons) that derive from a variety of biological and cellular processes. Therefore, they can provide crucial information about the genomic, epigenomic, matrix, and environmental outcomes of a cell, tissue, or organism, creating the perfect association between genes and their respective phenotypes. Since metabolites can be related to specific biological functions and processes in systems, cells, or tissues, a metabolic investigation may prove to be more advantageous than the study of genomics, transcriptomics, and proteomics [77,81,107,108,109]. Due to their medical and biological signature, these molecules permit the quantitative measurement of the dynamic chemical reactions that occur in living systems in response to a pathophysiological insult or genetic variation, and they can be accessed via biological matrixes such as blood, urine, plasma, serum, and FF [77,109]. The metabolomic analysis of FF revealed that lipid metabolites in PCOS patients are significantly altered [2]. Because the causes of infertility can be traced back to a metabolic imbalance, metabolomics can be used in reproductive medicine to identify and quantify low molecular weight metabolites found in the FF. The use of metabolomics made it possible to identify molecules found downstream of gene expression, providing critical information on cellular function [79]. All these factors make them attractive biomarker candidates, perfect for the study of human oocytes and embryos, as well as their development [77,109].

To date, the most used techniques used to analyze and measure FF constituents are nuclear magnetic resonance (NMR) [110,111,112,113], gas chromatography coupled with mass spectrometry (GC-MS) [114,115], liquid chromatography coupled with mass spectrometry (LC-MS) [2,84,116,117,118], and liquid chromatography with tandem mass spectrometry (LC-MS/MS) [1,119,120,121]. Some of the characteristics of these techniques are specified in Table 1. Some studies have also resorted to ultra-high performance liquid chromatography coupled with mass spectrometry (UHPLC-MS) [122], ultra-high performance liquid chromatography with tandem mass spectrometry (UHPLC-MS/MS) [76], multiple reaction monitoring (MRM)-profiling [123], and different types of immunoassays [33,124,125,126].

The data obtained through NMR presents high reproducibility. It permits several types of analyses but is essentially used for non-selective approaches, as it allows the identification of structures of unknown compounds belonging to different metabolic pathways and fluxes through the employment of isotope labels [129,130,131,132,142]. It facilitates metabolic studies in living organisms and can be used in vivo, permitting the analysis of metabolites present in biological fluids, cell extracts, and tissues without the need for fractionation techniques. The intensity of the signal generated is directly proportional to the metabolite concentrations and number of nuclei in the molecule, therefore enabling the quantification of more abundant compounds [129,130,131,132,142,143]. Compounds that are difficult to ionize or that require derivatization might be studied through this technique since it allows the identification of compounds with identical masses, including those with different isotopic isomers distributions. Due to stable isotope labels, NMR can also elucidate the dynamics and mechanisms of metabolite transformations and explore the compartmentalization of metabolic pathways in just one measurement [129,143]. These aspects made NMR one of the preferred platforms for long-term or large-scale clinical metabolomic studies [132]. The main disadvantage of this technique is its low sensitivity since the peaks of the various metabolites detected may overlap. However, it is possible to overcome this issue by performing multiple scans, using higher magnetic field strength, and cryo-cooled, microprobe, and hyperpolarization methods [129,130,131,132]. The most used nuclei in biomolecular NMR studies are 1H (proton), 13C, 15N, and 31P, with 1H being the most sensitive and most used in the study of metabolomics [129,144].

Metabolites can be measured using GC-MS [145,146]. These include several volatile compounds that can be measured directly and non-volatile or semi-volatile metabolites that can be accessed after derivatization. There is currently a wide range of instruments that vary in ionization and mass separation. The most common type of instrument is a single-quadrupole mass spectrometer with electron impact (EI) ionization [145]. When a sample stream collides with an electron beam, an electron is lost from the sample molecules, and the resulting ions represent the total mass of each analyte. The molecular ion usually fragments due to the large amount of energy imparted, producing smaller ions with characteristic relative abundances that provide a ‘fingerprint’ for that molecular structure [145,147,148,149,150]. A small sample is injected into the GC, vaporized, and directed to the chromatographic column by the carrier gas (typically helium) [145]. The GC column outlet is connected to the ion source of the MS via a heated transfer line, where the compounds eluting from the column are ionized [145,147,148,149,150]. The interactions between the carrier gas phase and the stationary phase caused the molecules in the sample to separate. To achieve the highest resolution, the capillary column should be selected in accordance with the sample [145]. LC-MS and LC-MS/MS are two techniques that are increasingly being used in routine analysis, specifically when referring to body fluid samples [151,152,153,154,155]. However, when establishing LC-MS screening procedures in conventional work, several limitations are presented. When compared to electron ionization mass spectra, the spectral information of electrospray ionization (ESI) and atmospheric pressure chemical ionization (APCI) spectra is limited. The formation of structure-related fragments arises from the collision-induced dissociation due to an increase in orifice or fragmentor voltage. Modern equipment allows rapid switching between different voltages, permitting distinct traces to be recorded during separation. As a result, compounds with different fragmentation properties can be analyzed in a single run without information loss. The degree of fragmentation varies greatly depending on the instrument [151,152,154,155,156,157,158,159,160,161]. Mutual ionization suppression or enhancement may affect quantitative LC-MS(/MS) assay accuracy, linearity, precision, and sensitivity. The presence of compounds in the spray chamber can change the efficiency of droplet formation or evaporation, affecting the amount of charged ions in the gas phase that eventually reaches the detector. Ion suppression is caused by drugs, endogenous compounds, ion-pairing agents, metabolites, isotope-labeled internal standards, and salts [151,162,163,164]. LC-MS, on the other hand, is an excellent complement to GC-MS for screening, library-assisted identification, and quantification of thermolabile, low-dosed, high-molecular, and polar compounds [151,152,153,154,155].

Although many biomarkers have been evaluated in the FF, there are a few consistent results among different works in the literature. Metabolites must be analyzed in the FF of the dominant follicle to reflect more accurately the dynamic changes in concentrations that occur during development [165]. The dominant follicle is the largest follicle, making it the clearest and also free from contaminations, such as blood cells [1]. The lack of consensus can be related to the criteria used in the analytical approaches, such as sample preparation of the FF, the presence of contaminants, and mass range. These might derive from the analysis of different follicular sizes, differences in methods for measuring analytes, patient age, number of patients, or number of analyzed samples, genetics, BMI, ovarian stimulation, infertility, or other disease diagnoses. Another reason may be linked to the heterogeneity in the IVF protocols, to the human ability to respond to IVF procedures, or even to the psychophysiological characteristics of each woman (or couple). Analyses taking place over different periods give rise to FFs with heterogeneous metabolic profiles, so accuracy also varies from study to study. Many of these aspects affect the content of follicle hormones and reflect the oocyte and embryo quality. Various external factors such as environmental pollution, smoking, heavy metals, or pesticides can increase levels of oxidants in the body and act as confounders. Most studies are limited, as even daily lifestyle and dietary patterns can influence the pathogenesis of PCOS and its biomarkers [1,3,33,45,113,120,166].

## 4. Biomarkers in Follicular Fluid

PCOS is one of the leading causes of female infertility and subfertility around the world, with many women going undiagnosed. The disorders involved in this pathology cause abnormalities in a variety of metabolic pathways, with many metabolites found to be deregulated. Because these biomarkers are associated with oxidative stress, lipidic and amino acid abnormalities, glucose metabolism discrepancies, hormonal disfunction, and inflammatory processes, several metabolomic approaches have been developed to study them and reveal their mechanisms of action. It is critical to assess and comprehend all these mechanisms and biomarkers in order to facilitate and enhance future treatments, making them more precise.

### 4.1. Biomarkers of Oxidative Stress

The antioxidant defense system has many components [124]. The imbalance between antioxidant and pro-oxidant molecules characterizes oxidative stress [80]. Healthy women present higher total antioxidant status (TAS) concentrations in the FF. These also have a positive association with clinical pregnancy rates. The TAS in FF samples can be determined using available immunoassays that measure the total antioxidant capacity of a sample [124]. The presence of various oxidative stress (OS) markers in human FF has been reported, and these appear to be involved in the pathogenesis of female infertility. An imbalance in the production of reactive oxygen species (ROS) could also be harmful. The effects of oxidative stress on female fertility are a topic of great interest, both scientifically and clinically. Several studies have found that follicular fluid ROS play a role in ovarian aging and, as a result, oocyte quality [80,98,167,168,169,170].

When accompanied by hyperglycemia, IR causes OS and lipid peroxidation, affecting steroidogenesis and follicular development. OS may be one of the causative factors of female infertility, originating many degenerative changes to the oocytes during aging [124]. There is a delicate balance of oxidants and antioxidants in an oocyte and its surrounding environment, and any disruption can compromise its competence. FF forms the immediate microenvironment of the developing oocyte and is the best medium to assess OS marks. Some studies showed that antioxidants increase dominant follicle selection and the cytoplasmic maturation of MII oocytes and improve embryo development. However, the correlation between ROS and the total antioxidant capacity (TAC) concentrations in the FF and embryo quality is not yet clear. It is possible to measure the levels of ROS, TAC, and lipid peroxidation [33].

**Figure 1:**
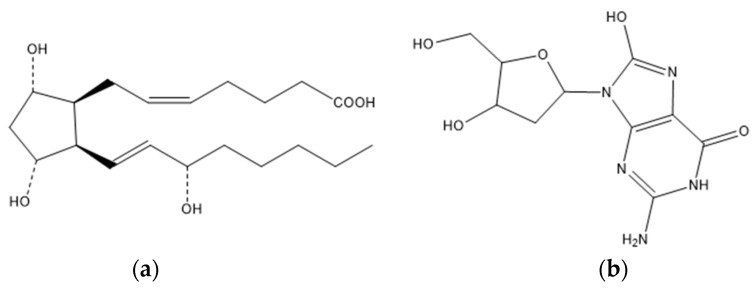
Biomarkers of oxidative stress: (**a**) chemical structure of 8-Isoprostane; (**b**) chemical structure of 8-hydroxy-2′-deoxyguanosine.

In addition, 8-Isoprostane (8-IP) (Figure 1a) is a highly sensitive, chemically stable, and quantifiable marker of OS in PCOS. It is a lipid peroxidation marker that can be measured using immunoassays. In addition, 8-IP is a prostaglandin F2-like compound formed by the free radical-catalyzed peroxidation of phospholipid-bound arachidonic acid, a pathway not involving cyclooxygenase. Its formation is modulated by antioxidant status, which increases in response to oxidant injury. Lipid peroxidation is caused by the free radical attack. Lipid peroxidation is a self-propagating phenomenon that is terminated by antioxidants, and the measurement of lipid peroxidation products is commonly used to assess OS. Women with PCOS have higher median 8-IP values in their FF. Pregnant women with PCOS who had carried abortions had significantly higher levels of 8-IP. As a result, 8-IP may predict a higher risk of miscarriage in PCOS women [33,124,171].

Fabjan and co-workers [124] found that 8-hydroxy-2’-deoxyguanosine (8-OHdG) (Figure 1b) in the FF is a good predictor of oocyte fertilization and maturation in PCOS patients. This OS biomarker concentration is significantly lower in these women. In addition, 8-OHdG is an oxidized deoxyguanosine derivative, one of the most common oxidative modifications in mutagenic damage. Guanosines are easily oxidized, and this reaction can result in G:C→T:A mutations. These mutations have the potential to cause serious consequences. Special DNA repair machinery typically recognizes and excises oxidized bases. High ROS levels stimulate the expression of antioxidant enzymes, reducing the extent of oxidative stress and, as a result, preventing ROS interactions with DNA and decreasing 8-OHdG formation. Several studies found that major antioxidant enzymes are significantly increased in PCOS patients [124,172,173,174]. The concentration of 8-OHdG in the PCOS group was linked to a mature egg and its successful fertilization. However, more research is required before 8-OHdG can be considered a PCOS biomarker [124].

### 4.2. Lipids

The FF’s composition includes diverse lipids, and diseases such as hyperandrogenemia, obesity, and IR are linked to lipid abnormalities. Meanwhile, obesity has a significant impact on lipid metabolism. Several biomarkers have shown that lipids are associated with molecular processes in normal-weight PCOS patients, such as inflammatory processes and endoplasmic reticulum stress in the FF. These will endanger oocyte nuclear maturation [2]. Free fatty acids are also considered critical molecular indicators, and several spectroscopy-based studies have found that the concentrations of these molecules in the FF of women with PCOS are also altered. Free fatty acids are produced as a result of abnormal lipid metabolism, and by modulating gene expression, they influence cell growth, differentiation, and metabolism. The composition of fatty acids in the oocyte and their concentration in the surrounding environment may impact oocyte developmental competence and subsequent embryo implantation in mammals. These abnormal levels of metabolites have been proposed to induce multiple endoplasmic reticulum stress markers that are harmful to mammalian oocytes [116]. As referenced before, lipid abnormalities can also be associated with IR. This specific condition might regulate the expression of glucose transporters in GCs, reducing glucose uptake in oocytes and delimiting the resources available for energy metabolism [112,175]. Defective glucose transportation and IR in PCOS patients induce alternative energy pathways that produce altered FF concentrations bioproducts such as lipids, amino acids, and ketone bodies [116].

Related to lipid metabolism, Chen et al. [122] studied the concentrations of 7β-Hydroxycholesterol (Supplementary Material Figure S1a) through LC-MS. 7-Hydroxycholesterol is an oxysterol, a bioactive metabolic intermediate and a product of cholesterol metabolism. Changes in 7-Hydroxycholesterol levels may cause oxidative stress, leading to fatty acid metabolism dysfunction. As a result, low levels of 7-Hydroxycholesterol in the FF of PCOS women may relate to a disrupted microenvironment for the growth of the oocytes. In addition, Ban and co-workers detected many species of phosphatidylethanolamines (PE) (Supplementary Material Figure S1b) in the FF of the PCOS patients using LC-MS [2]. PE is a major phospholipid class in the membranes of eukaryotic cells, creating a non-lamellar structure and modulating the membrane fluidity [2,176]. Cordeiro and their research team analyzed FF samples from PCOS patients who underwent IVF and had a hyper response to gonadotropins. Higher levels of PE were also characteristic of the PCOS group. This presence may be involved in the final process of cell division due to high proliferation in response to ovarian stimulation. The lipidomic analysis was performed by electrospray ionization mass spectrometry, and the biomarkers were analyzed by Electrospray Ionization MS/MS [176].

Phosphatidylinositol (PI) (Supplementary Material Figure S1c) is another subclass of lipids that is highly present in PCOS patients with average weight. PI presents a glycerol backbone, two esterified acyl chains, and an inositol ring linked by a phosphate that can also be detected by LC-MS. Although PI constitutes only 5–10% of total cellular lipids in mammalian cells, it is the source of seven phosphorylated derivatives that play vital roles in many cellular functions, such as signaling, membrane trafficking, ion channel regulation, and actin dynamics [2,177]. Thus, the detection of PE and PI may come from membrane structures of sub organelles or vesicles who suffered cell apoptosis [2]. Another study discovered low levels of 1H-Indol-3-ylacetyl-myo-inositol (Supplementary Material Figure S1d) through UHPLC-MS, another derivative from Indole [84].

Multiple reaction monitoring (MRM) metabolomic analysis of FF revealed that PCOS patients have a different lipid profile [123,166]. The presence of phosphatidylcholine (PC) (Supplementary Material Figure S1e) in human cumulus cells (hCC) was linked to LH from IVF cycles, implying that PC might relate to optimal oocyte development and proper LH response [166,178]. As a result, PCOS women have higher levels of LH in their FF [66,166].

Sphingolipids are cellular membrane structural components. They can function as signaling molecules, second messengers, or paracrine regulators of genetic transcription. These molecules also have the potential to regulate cell growth, proliferation, metastasis, apoptosis, senescence, immune responses, and chemo/radio-resistance [84,166,179]. The decrease of these compounds in PCOS patients’ FF might indicate changes in the proper process of steroidogenesis [166]. As the center lipid, ceramide (Supplementary Material Figure S1f) can be hydrolyzed from glucosylceramide (Supplementary Material Figure S1g) and synthesized from sphingomyelins (SM) (Supplementary Material Figure S1h), sphingosine, or galactosylceramide. Glycerophospholipid metabolism interconnects with the sphingolipid metabolism by the synthesis of SM from PC and ceramide [84,180]. Glucosylceramide was transferable to lactosylceramide, an interconnecting compound linked to glycosphingolipid biosynthesis [84,181]. Regarding the sphingolipid’s pathway, Liu and co-workers detected a decrease in ceramides, galabiosylceramide (Supplementary Material Figure S1i), glucosylceramide, SM, lactosylceramides, and tetrahexosylceramide (Supplementary Material Figure S1j) levels in the FF, using UHPLC-MS [84].

In the glycerophospholipid pathway, using UHPLC-MS and LC-MS, differences were observed between controls and PCOS patients. Lysophosphatidylcholines (lysoPCs) (Supplementary Material Figure S1k), lysophosphatidyl ethanolamines (lysoPE), and glycerophosphocholine were found to be up regulated in PCOS FF [84,106,116,122,181,182,183]. LysoPCs have been correlated with apoptosis, inflammation, and glucose regulation [84,184,185]. The reduced levels of PCs, phosphatidylglycerolphosphate (PGP) (Supplementary Material Figure S1l), lysophosphatidic acid (LPA) (Supplementary Material Figure S1m), and triglyceride (TG) (Supplementary Material Figure S1n), also detected by UHPLC-MS, lead to lower fertilization rates. TG acts as an energy supplier and tends to be present in high concentrations due to the oocyte maturation process [84]. PGP is an important precursor of cardiolipin, which is present in the mitochondrial membrane. Glycerol biosynthesis may arise from high lipolysis that occurs due to oocyte maturation, later originating TG and PGP [84,186]. Liu and their research team observed that LysoPE, lysoPC, and PC were highly associated with age and BMI. There was a positive correlation between lysoPE and the internal secretion parameter LH/FSH in all FF samples, while there was a significantly positive correlation of PC and LH/FSH [84].

Increased concentrations of glycerolipids containing stearic acid residues (Supplementary Material Figure S1o) in the FF of PCOS patients, on the other hand, might connect to the IVF clinical findings. The ovaries of IVF patients synthesize a large amount of estradiol. Its esterification by the E2-acyl-CoA acyltransferase produces stearates, which are then metabolized into fatty acid esters of estradiol [123,187]. According to studies, fertilization failed human oocytes contain more stearic acid than palmitic, oleic, linoleic, and eicosapentaenoic acids (Supplementary Material Figure S1p–s, respectively). The concentrations of oleic and stearic acid are associated with oocyte developmental competence, which may account for the decreased pregnancy rate in women with PCOS [112,116,123,188,189,190]. Previously, a GC/MS metabolomics approach involving PCOS and IVF demonstrated the presence of different fatty acids in the FF. An increase in palmitoleic and oleic acids was correlated with embryo fragmentation, deficient developmental competence of embryos, and consequent poor IVF pregnancy outcomes [30]. However, through multiple reaction monitoring (MRM)-profiling, Cordeiro and co-workers did not find oleic acid as a compound of increased abundance, and palmitoleic acid was related to better outcomes [123]. Sun and collaborators resorted to LC-MS to study the concentrations of both acids, finding higher quantities in the FF of obese PCOS. They also found low FF levels of lysoPCs and phytosphingosine (Supplementary Material Figure S1t), and high levels of eicosapentaenoic acid [116]. Liu and colleagues discovered high levels of pyruvic, citric, isocitric, stearic, and palmitic acid using GC/MS [115]. The levels of lithocholic and sinapinic acid, on the other hand, were significantly lower. Pyruvate and isocitric acid are crucial intermediates in the tricarboxylic acid cycle, a metabolic pathway that involves sugar, lipid, and amino acid metabolism [4,115,191,192]. Sinapinic acid is a cinnamic acid derivative with strong anti-diabetic properties that can also prevent the formation of hydroperoxides by preventing lipid oxidation. As a result, the significant decrease in sinapinic acid may be related to IR, as well as abnormal lipid metabolism, the tricarboxylic acid cycle, amino acid biosynthesis, the glucagon metabolic pathway, and fatty acid biosynthesis, all of which have a significant impact on metabolic changes in PCOS patients with IR [4,59,193].

Reduced triglyceride (TG) levels in the FF are strongly linked to lower fertility rates in PCOS. Increased BMI, on the other hand, is correlated with FF presenting higher TG levels. So far, TGs are the lipid subclass that presents the largest discrepancies between healthy and PCOS women. TGs are composed of three fatty acids and glycerol, a crucial power supply. Patients with PCOS frequently have dyslipidemia, presenting high LDL and TG and low HDL levels [2,194]. TGs in the FF were also associated with high levels of adipokines and proinflammatory cytokines, implying inflammatory processes. As a result, increased TG levels may correlate to poor oocyte quality in PCOS patients. TG accumulation in the FF was also correlated with high adipokines and proinflammatory cytokines, implying inflammatory processes. Therefore, increased TG levels might be associated with the low quality of oocytes in PCOS patients. These were determined by LC-MS [2].

Li and co-workers performed LC-MS to study the concentrations of another fatty acid, arachidonic acid (AA) (Supplementary Material Figure S1u), and its metabolites. The aim of this study was to decipher the role of local AA metabolism in the FF of non-obese PCOS patients that underwent IVF [117]. Some studies related to non-PCOS patients had already stated that high levels of AA in the FF were detrimental for oocytes [117,195]. The levels of AA metabolites generated via cyclooxygenase-2 (COX-2) (PGI2, PGE2, PGD2, PGF2α, TXB2, PGJ2, and 15d-PGJ2) and cytochrome P450 epoxygenase (8,9-DHET and 11,12-DHET) pathways, but not lipoxygenases, were significantly higher. The metabolites generated via the COX-2 network were significantly correlated with the levels of testosterone and fasting insulin in serum. Insulin played a crucial role in the increased AA metabolites generated via COX-2, which could be interpreted as another novel molecular pathophysiological mechanism of PCOS [117]. AA-derived metabolites, especially prostaglandins (PGs), play key roles in female fertility. PGE2, PGF2α, and PGJ2 were found to be elevated in the FF of PCOS women. PGE2, an autocrine and paracrine mediator, boosts the release of luteinizing hormone-releasing hormone (LHRH). It affects oocyte maturation, cumulus expansion, and cumulus-oocyte coupling. When present in high concentrations, it might also be damaging, delaying follicle maturation. PGF2α is critical for ovulation once it increases collagenolysis and ovarian contractility. The increase in the PGF2α level in the ovary may serve to overcome the inability to ovulate properly in patients with PCOS [117,196,197,198]. PGJ2 levels are directly correlated with serum insulin and testosterone. Since PGJ2 is not stable in vivo, it is converted to cyclopentenone PGs such as 9-deoxy-Δ9,12,13,14- dihydro PGD2 (Δ12-PGJ2), and 15d-PGJ2 [117,199]. The last one is an endogenous ligand of peroxisome proliferator-activated receptor gamma that acts as an inflammatory regulator and controls GCs proliferation, steroid hormone biosynthesis, and fibrosis [117,200,201,202,203,204]. Many studies have tried to find a correlation between the role of insulin on COX-2, but so far without success. Insulin can increase COX-2, IL-1β-induced COX-2, and PGE2 production [205,206]. However, some studies stated opposite relations, with insulin decreasing COX-2 expression [207,208]. Therefore, the AA metabolites in the FF specifically reflect local ovarian state. It is of notice that gonadotropin stimulation also upregulates PGs levels. Hyperinsulinemia could also exaggerate the induction role of inflammation, stimulating GCs to produce more PGs [117].

### 4.3. Amino Acids

Regarding the amino acid metabolism, through MS/MS [115], LC-MS [116], and UHPLC [122] there were detected high levels of valine (Supplementary Material Figure S2a), isoleucine (Supplementary Material Figure S2b) [115], leucine (Supplementary Material Figure S2c) [115,116], phenylalanine (Supplementary Material Figure S2d) [116], D-glutamic acid (Supplementary Material Figure S2e), ferulic acid (Supplementary Material Figure S2f), lysine (Supplementary Material Figure S2g), salicylic acid (Supplementary Material Figure S2h), and 3-methylhistidine (Supplementary Material Figure S2i) [122]. Additionally, through NMR [110], low levels of alanine (Supplementary Material Figure S2j), tyrosine (Supplementary Material Figure S2k), and glutamine (Supplementary Material Figure S2l) were registered [115]. Glutamine presents a highly positive correlation with top-quality rate embryos, and its decrease may lead to fertilization failure [110,209]. As well as valine, isoleucine, and leucine, other amino acids belonging to the branched-chain amino acids catabolism were found relatively high, such as α-keto-β-methylvalerate (Supplementary Material Figure S2m), α-ketoisovalerate (Supplementary Material Figure S2n), and α-ketoisocaproate (Supplementary Material Figure S2o) [115]. Contrary, Iaccarino and colleagues demonstrated, through 1H NMR spectroscopy, that hyperinsulinemic PCOS women presented higher levels of alanine [113], and Castiglione Morelli and co-workers detected decreased levels of leucine in the metabolomic analysis of FF, performed by NMR spectroscopy [112]. Follicular androgenization can cause metabolic differences in women with PCOS, which can then be linked to IR. This could be due to a decrease in leucine levels as well as an increase in glycerol and lipid levels, both of which are common indicators of altered lipolysis [112,210,211]. Insulin sensitivity appears to regulate the expression of glucose transporters in GCs, which may control oocyte competence by reducing oocyte glucose uptake and, thus, the resources available for energy metabolism. As a result, compensatory mechanisms in PCOS oocytes could alter FF composition, negatively impacting physiological oocyte maturation and quality [112,175,212]. PCOS patients present elevated numbers of oocytes, even if they are not of the best quality. This growth requires abundant protein supply, and glutamic acid works as an alternative source of energy. This could explain the increased levels in the samples. However, protein synthesis might be affected [122,213,214].

Through UHPLC, Chen and collaborators studied specific metabolic pathways related to PCOS, such as the vitamin B6 metabolism, phenylalanine metabolism, and carnitine synthesis [122]. D-Glutamic acid and Pyridoxal 5′-phosphate (PLP) (Supplementary Material Figure S2p) were identified as products of the vitamin B6 metabolism [122]. They are involved in glycogen and amino metabolic pathways and in the synthesis of neurotransmitters, such as D-serine [122,193,215,216,217,218,219]. PLP, an essential enzymatic cofactor, is abundant in PCOS patients. PLP-dependent enzymes participate in critical cellular processes such as amino acid and amine biosynthesis and degradation, as well as in cardiovascular, hematopoietic, immune, and metabolic systems. In addition, PLP is a necessary coenzyme for the metabolism of homocysteine and participates in carnitine synthesis. Its disruption might compromise the oocyte microenvironment. To grow, oocytes depend on glutamic acid to fulfill their protein necessities. The metabolism of vitamin B6 provides a cofactor for the enzyme glutamate decarboxylase, which modifies glutamic acid levels. In PCOS, glutamic acid acts as an alternative energy source, influencing protein synthesis. As a result, D-glutamic acid up-regulation in PCOS may indicate a decrease in oocyte utilization and subsequent accumulation in the FF, affecting its growth. Phenylalanine metabolism is crucial for the growth and development of the oocyte and has been linked to ovulatory dysfunction in women [118,122,213,214,220,221,222,223,224,225,226,227,228,229]. Elevated levels of salicylic acid are involved in phenylalanine metabolism [122].

From the metabolic pathway of fatty acids, 3-hydroxynonanoyl carnitine, L-Carnitine (Supplementary Material Figure S2q), and eicosatetraenoic acid (Supplementary Material Figure S2r) were found to be up regulated, according to the LC-MS and UHPLC Q-Exactive analysis. However, acylcarnitines (Supplementary Material Figure S2s) were found down regulated [106,116,122]. L-Lysine, which was stated before as being up regulated, is also involved in the synthesis of carnitine. L-Carnitine is very relevant for the carnitine synthesis pathway, transporting fatty acids through the mitochondrial membranes and to the mitochondria. These will lead to an increase in the fatty acid’s oxidation and glucose metabolism. Mitochondrial dysfunction may also be present in PCOS patients. The increase in L-Carnitine in PCOS comes from a decreased utilization and subsequent accumulation in the FF [122].

### 4.4. Glucose and Derivatives

**Figure 2:**
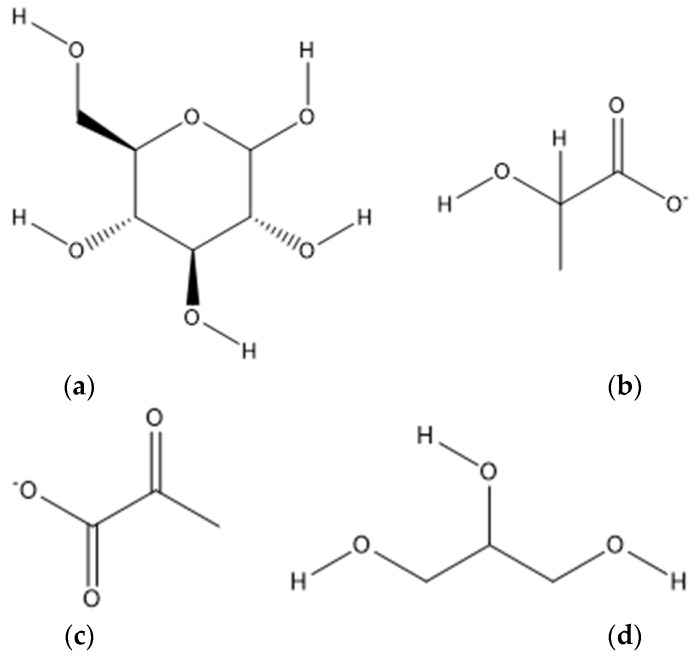
Glucose and derivatives: (**a**) chemical structure of glucose; (**b**) chemical structure of lactate; (**c**) chemical structure of pyruvate; (**d**) chemical structure of glycerol.

Glucose (Figure 2a), lactate (Figure 2b), and pyruvate (Figure 2c) are metabolites that belong to the glycolytic pathway, and they are, for that reason, strongly related [84,110,113,115,122]. The most widely used compounds by the oocyte to produce energy are lactate and pyruvate [115,230]. Specifically, granulosa cells (GCs) are avascular and present a thick layer, causing the follicles to grow in anaerobic conditions [113,231]. The oocyte is also incapable of performing glycolysis, making GC and CC essential, once they are provided products from this pathway [113,232]. Pyruvate is the product of glycolysis, and its transformation into lactate is obtained through the limited amounts of oxygen available. When transferred to the oocyte, pyruvate cannot be converted to lactate, one of the main compounds produced by the GC [113,115]. High levels of glucose were detected in the FF of PCOS patients using NMR spectroscopy [112], and through UHPLC-MS and NMR spectroscopy, increased concentrations of glycerol (Figure 2d) were also registered, a very common feature of altered lipolysis caused by IR since it is also part of the synthesis of triacylglycerols and phospholipids [84,110,112]. Through NMR spectroscopy and UPLC-MS, lactate levels were found to be decreased in the FF of women with PCOS [84,110,112,115]. These might lead to crucial alterations in the epigenetic modifier function, since this metabolite is an inhibitor of histone deacetylases (HDACs), playing an important role in the gene expression of transcriptional regulation [115,233]. The hormone β-hCG, produced in great quantities during pregnancy, was negatively correlated with lactate [112,210]. According to Zhang and co-workers, pyruvate was also found significantly decreased in the RMN analysis [110]. However, Zhao and collaborators, and Sun and co-workers obtained the inverse results using MS/MS, indicating that women with PCOS present increased glycolytic activity to compensate the energy supply [115,182,234,235]. It was stated that, between different PCOS women, hyperinsulinemic ones presented higher concentrations of lactate and pyruvate [113]. Through 1H NMR spectroscopy, Iaccarino and team recorded negative correlations between glucose and lactate and among glucose and pyruvate, and positive ones were between pyruvate and lactate [113]. Indeed, additional studies have already showed that PCOS women presented an inverse relationship between lactate/pyruvate ratios [115,230]. These data were in agreement with metabolite correlations found in the FF of women without PCOS [113,236]. Thus, the strong link between glucose, pyruvate, and lactate confirms that the FF provides lactate and pyruvate to the developing oocyte as a source of energy. It also suggests that an inefficient glucose mechanism in granulosa-lutein cells is harmful [110,237] and how important anaerobic metabolism is for follicle development [113,231].

### 4.5. Steroid Acids

Bile acid metabolism is abnormal in PCOS patients. Its concentrations and metabolites are found to be higher in these women’s FF. Bile acid, a constituent of bile, is biosynthesized by the liver and decomposed into bile acid derivatives by intestinal flora. They become conjugated bile acids when combined with glycine or taurine. Bile acids have an endocrine function, are involved in glucose and lipid metabolism, and have some influence on the pathogenesis of diabetes, all in addition to regulating their synthesis. They are cytotoxic and can harm the ovarian microenvironment and function in PCOS patients by damaging mitochondrial function and inducing oxidative stress, resulting in the growth and development of follicular and ovulation disorders [4,76]. The entire synthesis pathway of bile acid is present in follicles, implying that bile acid might be produced by GCs [76,238]. Human FF presents high levels of bile acids, almost twice that of serum, and ursodeoxycholic acid, a secondary bile acid, resulting in high-quality embryos. Some research has suggested that regulating bile acid transport from blood to the FF may influence the reproductive system. Farnesoid X receptors (FXR), a natural bile acid receptor, are found in ovarian GCs. These may have an influence on ovarian function by regulating the function of GCs. Liver receptor homolog 1 (LRH-1) plays a role in gonadal development and ovulation as an essential component of bile acid metabolism. PCOS patients’ ovarian GCs also have high LRH-1 expression. All this evidence suggests that bile acid metabolites may be involved in follicular development and ovarian function in PCOS [76]. Yang and co-workers highlighted four specific conjugated bile acids, resorting to a bile acid immunoassay and a UHPLC-MS/MS method: glycocholic acid (GCA) (Supplementary Material Figure S3a), taurocholic acid (TCA) (Supplementary Material Figure S3b), glycochenodeoxycholic acid (GCDCA) (Supplementary Material Figure S3c), and chenodeoxycholic acid-3-β-d-glucuronide (CDCA-3Gln) (Supplementary Material Figure S3d). Generally, the LH signal is closely related to ovulation, implying that the elevated concentration of GCDCA in the FF is associated with PCOS ovulation disorders. Meanwhile, CDCA-3Gln levels were positively correlated with AFC, indicating that CDCA-3Gln may be related to the ovarian reserve [76].

### 4.6. Gonadotrophines

A low FSH/LH ratio is a common endocrine disturbance in PCOS patients, and it is associated with an increase in LH and a decrease in FSH levels. This will influence the production of follicles and oocytes, as well as their maturation during IVF cycles. Because a decrease in the FSH/LH ratio is associated with an increased follicle and oocyte production in PCOS, this hormonal imbalance affects the metabolomic profile of the FF, resulting in oocyte and embryo impairment [70,166,239]. PCOS is associated with a high number of embryos. However, when compared to controls, these are of lower quality, a condition that worsens with age. These clinical parameters may be related to lower levels of specific fatty acids, such as oleic and stearic acids. When compared to controls, patients with PCOS have a higher number of follicles and, as a result, a higher number of oocytes. Nevertheless, the rate of oocyte retrieval is lower in PCOS patients [166].

### 4.7. Steroids and Other Hormones

**Figure 3:**
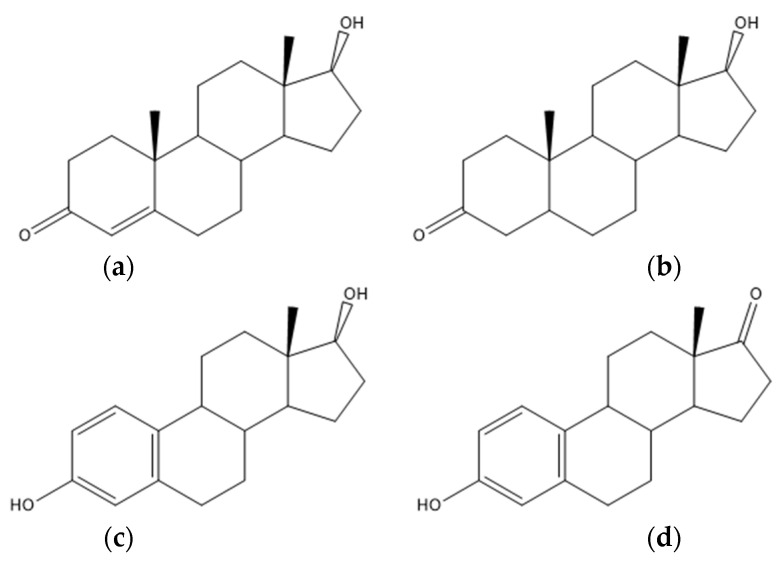

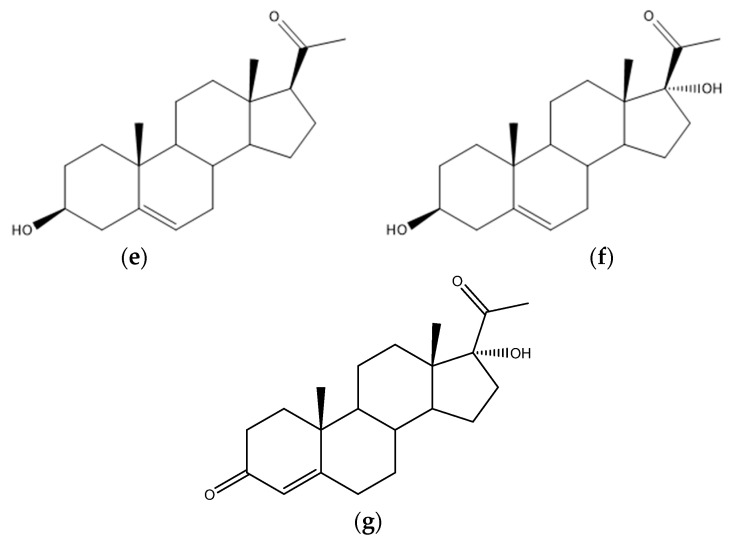
Steroid hormones: (**a**) chemical structure of testosterone; (**b**) chemical structure of dihydrotestosterone; (**c**) chemical structure of estradiol; (**d**) chemical structure of estrone; (**e**) chemical structure of pregnenolone; (**f**) chemical structure of 17-Hydroxypregnenolone; (**g**) chemical structure of 17-Hydroxyprogesterone.

The bioactive steroids present in the FF play vital roles in regulating ovarian folliculogenesis and oocyte maturation. Variations in androgens (testosterone, dihydrotestosterone, estrogens, estrone (Figure 3a–d, respectively)) or progesterone levels might compromise the occurrence of healthy oocytes, as well as their arrest [1,76]. Compared with the FF of healthy patients, the FF of PCOS women presented higher androgen and lower estrogen levels [76]. Women whose pathology was detected at an early age due to abnormal ovulation or menstruation, and were correctly monitored, did not show as many complications and presented higher fertilization rates [31,84,240].

It was suggested that ovaries are the leading source of androgen excess in the FF of PCOS women [120]. Abnormal androgen levels induce many characteristic features of PCOS, including hirsutism, acne, ovulatory dysfunction, polycystic ovarian morphology, and even metabolic disturbances. This increase can also generate intra-ovarian androgen excess. However, not all PCOS women are hyperandrogenic, and there are discrepancies between different ethnic populations [1]. P450c17 and 3βHSD2 are key upstream enzymes of androgen synthesis, and both reveal increased activity in PCOS individuals. Meanwhile, P450aro and 5α-reductase have reduced activity. These findings might help to explain the androgen excess in PCOS, consistent with and expanding the discoveries of earlier reports [1,45,241,242]. The FF samples from PCOS women presented high levels and an decreased activity of 11βHSD2, an enzyme that catalyzes the conversion of biologically active cortisol to inactive cortisone [1,241].

Hyperandrogenism, caused by steroidogenesis dysregulation, is central to the pathogenesis of PCOS. The altered expression of critical enzymes in the steroidogenic pathway is responsible for ovarian steroidogenesis patterns [1]. Hyperandrogenism is also characterized by nonclassical congenital adrenal hyperplasia and hyperprolactinemia [3]. Through LC-MS, Sun and colleagues discovered that deoxycorticosterone levels were higher in the FF of PCOS women, influencing steroid hormone biosynthesis. The increase in FF deoxycorticosterone may be due to adrenal steroid metabolites, modulated by selective adrenal factors. The overresponse of adrenal androgen to ovarian stimulation protocol drugs and increased hydroxylation activity could be the causes of the excess adrenal androgen observed in these patients [1,116,120].

Estradiol levels in the FF are higher in PCOS women [1,112,189,190]. During follicular development, the dominant follicle is the primary site of estrogen production. Estrogen synthesized by GCs regulates cell replication and cytodifferentiation in response to FSH and LH endocrine stimulation [1]. Yang and collaborators performed LC–MS/MS and registered low follicular levels of E2 (estradiol) and testosterone (T) in the FF of patients with PCOS. Overall, these women showed generally decreased steroidogenesis [1,120]. Yu and colleagues discovered that estrogen and pregnenolone (Figure 3e) are abundant in these samples and may influence follicle development in PCOS patients using LC–MS/MS. The mechanism could be linked to changes in HSD17B1, CYP19A1, and CYP11A1 expression in FF exosomes, which encode enzyme-induced steroidogenesis abnormalities. In steroidogenesis, follicular development, and some pathological conditions, exosomes act as message transmitters in intercellular communication, transferring substances such as proteins, lipids, miRNAs, and circRNAs. HSD17B1 and P450 aromatase (CYP19A1) expression is linked to follicular differentiation and elevated follicular E2 concentrations [1]. In contrast, via UHPLC and LC-MS/MS, Chen and research team and Yang and co-workers, respectively, registered a decrease in pregnenolone, 17-Hydroxypregnenolone (17-OH5P) (Figure 3f), and 17-Hydroxyprogesterone (Figure 3g) [120,122].

In addition, Naessen and collaborators measured several steroids by LC-MS/MS methods in the FF of PCOS patients and observed high levels of androgens and low levels of estrogens [45]. The elevated activity of CYP17-linked enzymes increases FF androgen levels [45,242,243,244], while the reduced activity of the ovarian CYP19- linked enzyme (aromatase) lowers FF estrogens in women with PCOS [45,241]. Of androgen precursors, only high levels of 17-OH5P were found in the FF from patients with PCOS. The first step of CYP17 conversion, where pregnenolone is transformed into 17-OH5P, is the only independent and significant predictor regarding the prevalence of PCOS, since it leads to higher levels of dehydroepiandrosterone, androstenedione, and testosterone. Diminished CYP19 activity (aromatase) contributes to the accumulation of A and T [45]. GCs of women with PCOS have their aromatase conversion activity compromised, due to the exposure to the altered FF from these patients, decreasing estrogen’s biosynthesis [45,245]. This may lead to anovulation and disable the classic follicular arrest. It is proposed that some of these abnormalities towards the PCOS hormonal status might regulate with advancing age. Indeed, there is an adaptation from the organism, normalization of symptoms of hyperandrogenism, and posterior regulation of menstrual periods [45].

The anti-Müllerian hormone (AMH) is present in high concentrations in the FF of PCOS women and can be detected by immunoassays. This hormone is a member of the transforming growth factor-β superfamily, and its levels in the FF are associated with ovarian reserve and follicular maturation rates. AMH is an excellent marker of ovarian reserve production, since its formation starts in very small follicles and continues to be produced by the GCs. AMH affects oocyte development during folliculogenesis, and its levels in the FF may affect the oocyte and embryo quality. There is a peak in its production, followed by a rapid decline. It might be used as a fertilization predicter since FF with higher concentrations of AMH generates more fertilized oocytes. AMH production within the follicles is the part of the mechanism responsible for selection of the pre-ovulatory follicle [113,124,246,247,248]. Using 1H NMR spectroscopy, Iaccarino and their research group noticed that normoinsulinemic PCOS women show even higher concentrations of AMH [113]. AMH and normoinsulinemic women have both been negatively correlated with BMI [113,249]. Lastly, AMH was positively correlated with glucose and negatively correlated with lactate, pyruvate, and alanine [113]. Data between its FF levels and ART outcomes in PCOS women are limited, contradictory, and primarily obtained from Long-GnRH agonist cycles. High levels of AMH in the FF inhibit ovarian folliculogenesis during various protocols regarding different GnRH agonist concentrations. The different patterns of follicular angiogenesis, on the other hand, affect the dependence of oocyte yield on AMH FF levels [250].

### 4.8. Interleukines

Chemiluminescence data from Z. Li’s research group revealed that the leukemia inhibitory factor (LIF) concentrations in the FF were decreased in PCOS patients and that its concentrations were negatively correlated with estradiol levels. Therefore, abnormal levels of LIF might lead to ovarian dysfunction and folliculogenesis disorders in PCOS patients [165]. LIF, a secretory glycoprotein member of the IL-6 family, has been associated with infertility and recurrent abortion. It can inhibit the differentiation and maintain the proliferation of embryonic stem cells, once it interferes with follicular development, maturation of oocytes, embryo implantation, growth, and differentiation [251]. LIF can be produced locally by the ovary or macrophages and in GCs present in the FF of women [165].

## 5. Discussion (Perspectives)

PCOS is a disease with a large global impact, causing infertility and preventing many couples from conceiving. There has been a significant increase in the number of articles and studies aimed at filling existing knowledge gaps in recent years. Due to significant scientific and technological advances, it is now possible to detect this pathology at an early stage, improving the prognosis of affected women and allowing for ART. The in-depth study of FF allows the analysis of a wide range of metabolites from different pathways. As a result, this biological matrix opens many doors to the understanding of PCOS and aids in the discovery of new biomarkers. However, successful and adequate therapies to mitigate all the negative effects of PCOS have yet to be developed, and further research in various fields of health sciences is required to uncover this disease and all its peculiarities. This study aims to frame and group the most recent metabolites discovered and studies in FF that are linked to PCOS.

## Figures and Tables

**Table 1:** Overall characteristics applied for the molecular analysis of FF.

Technique	Advantages	Disadvantages	References
NMR	Highly reproducible; Minimal sample handling and non-destructive; Identification of a wide range of low-molecular-weight compounds; Used for non-selective approaches.	Low sensitivity due to overlapped peaks; Requires expensive deuterated solvents; Suppression of residual protonated solvents; Difficult to find compatibility between the volume of the chromatographic peak and the volume of the flow cell.	[113,127,128,129,130,131,132,133]
GC-MS	Presents well-established libraries of both commercial and ‘in house’ metabolite databases available; Highly used for metabolite profiling and quantification.	Requires derivatization; Long analysis time; Does not allow real-time analysis or direct quantitative determinations; Presents a limit of sample capacity.	[134,135,136,137,138]
LC-MS	Wide metabolite coverage that presents high sensitivity and specificity; Versatility technology.	Untargeted screening is highly challenging; Lack of reference libraries.	[139,140,141]
LC-MS/MS	Higher specificity and selectivity; Gives more structural information.	Lack of reference libraries.	[139,140,141]

## Data Availability

This systematic search was performed via the databases Web of Science, SCOPUS, b-on, and PubMed. The key words and respective mergers used in the study were “follicular fluid” AND “metabolomics”, “metabolomics” AND “PCOS,” “follicular fluid” AND “PCOS”, and finally “PCOS” AND “follicular fluid” AND “PCOS”. These had to be included in the article’s title, abstract, or key words. The research period ranged from 2014 to 2021. The studies considered were all human-related, with participants ranging in age, physical and metabolic characteristics, and ethnicity.

## Supplementary Materials

The following supporting information can be downloaded at: https://www.mdpi.com/article/10.3390/biomedicines10061254/s1, Figure S1. Lipids: (a) chemical structure of 7β-hydroxycholesterol; (b) chemical structure of phosphatidylethanolamines; (c) chemical structure of phosphatidylinositol; (d) chemical structure of 1H-indol-3-ylacetyl-myo-inositol; (e) chemical structure of phosphatidylcholine; (f) chemical structure of ceramide; (g) chemical structure of glucosylceramide; (h) chemical structure of sphingomyelins; (i) chemical structure of galabiosylceramide; (j) chemical structure of tetrahexosylceramide; (k) chemical structure of lysophosphatidylcholines; (l) chemical structure of phosphatidylglycerolphosphate; (m) chemical structure of lysophosphatidic acid; (n) chemical structure of triglycerides; (o) chemical structure of stearic acid; (p) chemical structure of palmitic acid; (q) chemical structure of oleic acid; (r) chemical structure of linoleic acid; (s) chemical structure of eicosapentaenoic acid; (t) chemical structure of phytosphingosine; (u) chemical structure of arachidonic acid. Figure S2. Amino Acids: (a) chemical structure of valine; (b) chemical structure isoleucine; (c) chemical structure of leucine; (d) chemical structure of phenylalanine; (e) chemical structure of D-glutamic acid; (f) chemical structure of ferulic acid; (g) chemical structure of lysine; (h) chemical structure of salicylic acid; (i) chemical structure of 3-methylhistidine; (j) chemical structure of alanine; (k) chemical structure of tyrosine; (l) chemical structure of glutamine; (m) chemical structure of α-keto-β-methylvalerate; (n) chemical structure of α-ketoisovalerate; (o) chemical structure of α-ketoisocaproate; (p) chemical structure of Pyridoxal 5′-phosphate; (q) chemical structure of L-Carnitine; (r) chemical structure of eicosatetraenoic acid; (s) chemical structure of acylcarnitines. Figure S3. Steroid acids: (a) chemical structure of glycocholic; (b) chemical structure of taurocholic acid; (c) chemical structure of glycochenodeoxycholic acid; (d) chemical structure of chenodeoxycholic ac-id-3-β-d-glucuronide.
