# Supplementary material for: Follicular Fluid: A Powerful Tool for the Understanding and Diagnosis of Polycystic Ovary Syndrome
Source: Biomedicines. 2022 May 27;10(6):1254. doi: 10.3390/biomedicines10061254 (PMC9219683; doi:10.3390/biomedicines10061254)
Supplement: Supplementary file 1 [file biomedicines-10-01254-s001.zip › biomedicines-1733189-supplementary.pdf]

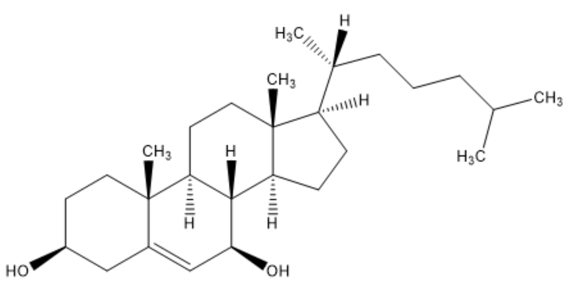

(**a**)

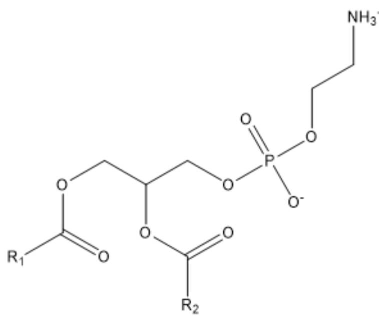

(**b**)

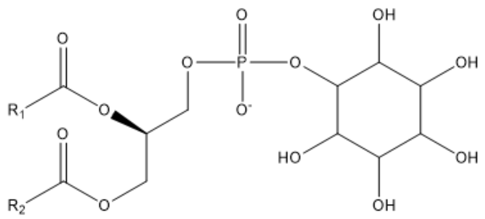

(**c**)

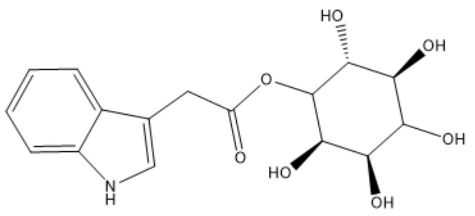

(**d**)

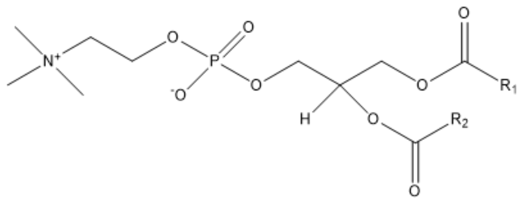

(**e**)

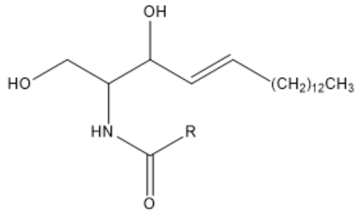

(**f**)

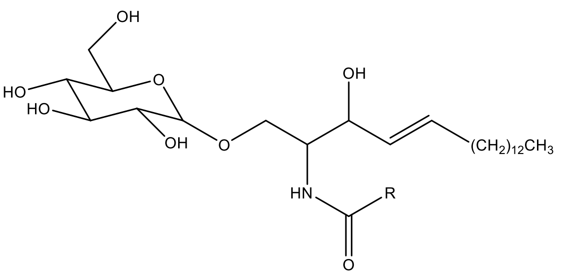

(**g**)

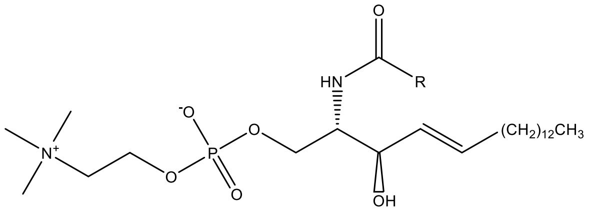

O
HN
R
-O
O
N+
O
P
O
(CH2)12CH3
O
OH

(**h**)

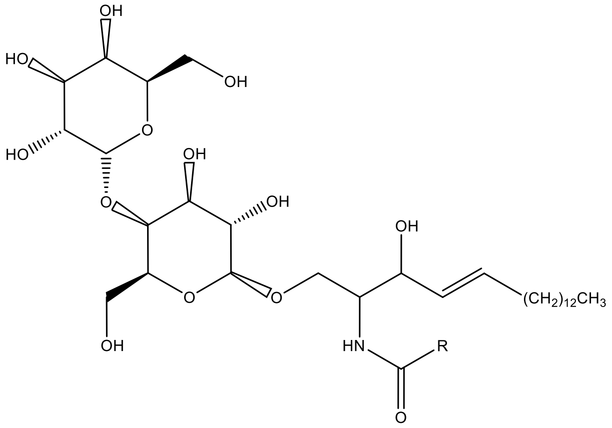

OH
HO
OH
HO
O
OH
O
OH
OH
O
O
(CH2)12CH3
OH
HN
R
O

(**i**)

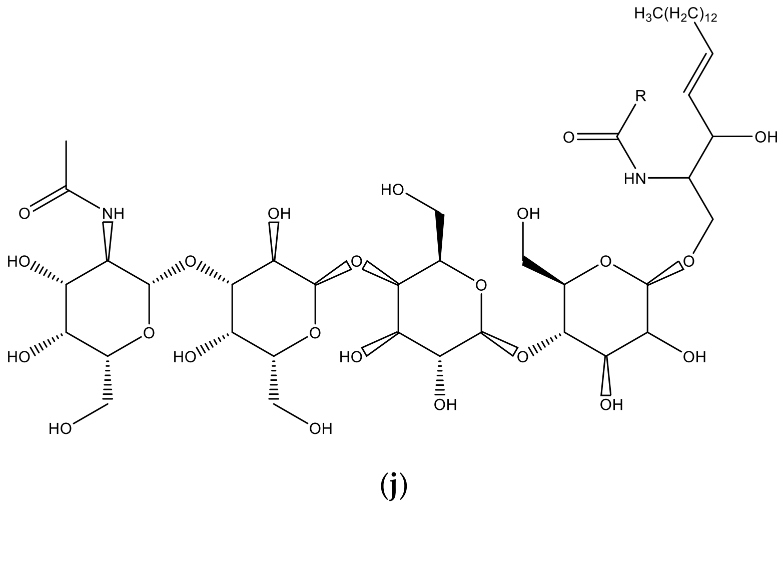

H3C(H2C)12
R
O
OH
HN
HO
OH
O
NH
OH
OH
HO
O
O
O
O
HO
O
O
HO
O
OH
HO
OH
HO
OH
OH
HO
OH

(**j**)

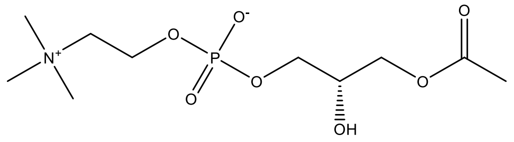

O⁻
N⁺
O
P
O
O
O
O
O
OH

(**k**)

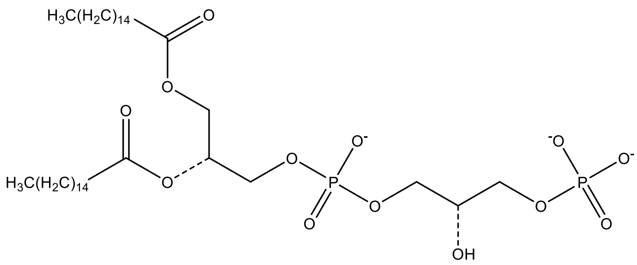

$H_3C(H_2C)_{14}$
$H_3C(H_2C)_{14}$
OH

(**l**)

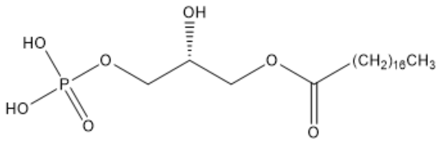

HO
HO
OH
$(CH_2)_{16}CH_3$

(**m**)

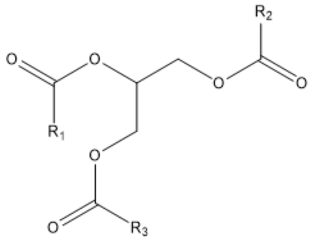

R_1
R_2
R_3

(**n**)

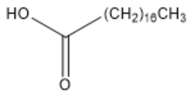

HO
$(CH_2)_{16}CH_3$

(**o**)

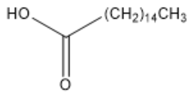

HO
$(CH_2)_{14}CH_3$

(**p**)

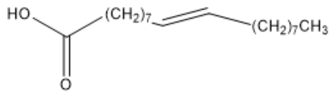

HO
$(CH_2)_7$
$(CH_2)_7CH_3$

(**q**)

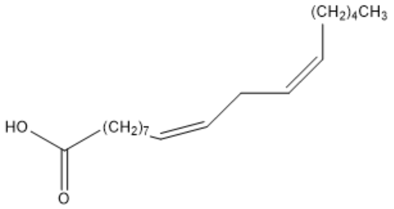

(**r**)

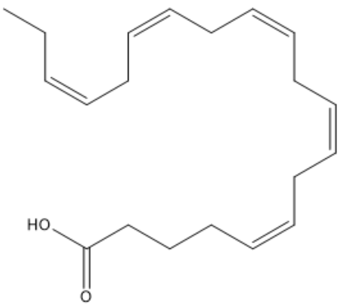

(**s**)

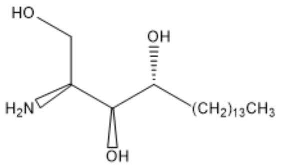

(**t**)

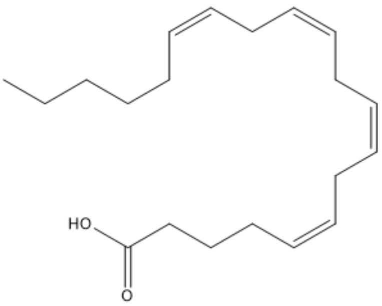

(**u**)

Figure S1. Lipids: (a) chemical structure of 7β-hydroxycholesterol; (b) chemical structure of phosphatidylethanolamines; (c) chemical structure of phosphatidylinositol; (d) chemical structure of 1H-indol-3-ylacetyl-myo-inositol; (e) chemical structure of phosphatidylcholine; (f) chemical structure of ceramide; (g) chemical structure of glucosylceramide; (h) chemical structure of sphingomyelins; (i) chemical structure of galabiosylceramide; (j) chemical structure of tetrahexosylceramide; (k) chemical structure of lysophosphatidylcholines; (l) chemical structure of phosphatidylglycerolphosphate; (m) chemical structure of lysophosphatidic acid; (n) chemical structure of triglycerides; (o) chemical structure of stearic acid; (p) chemical structure of palmitic acid; (q) chemical structure of oleic acid; (r) chemical structure of linoleic acid; (s) chemical structure of eicosapentaenoic acid; (t) chemical structure of phytosphingosine; (u) chemical structure of arachidonic acid.

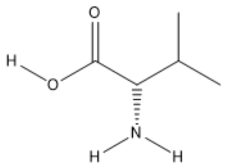

(**a**)

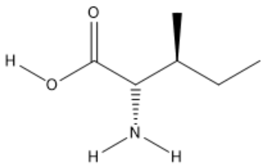

(**b**)

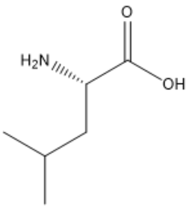

(**c**)

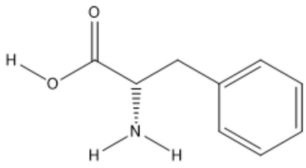

(**d**)

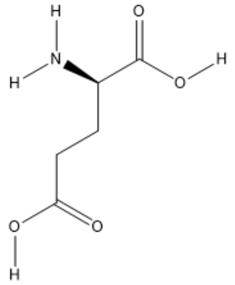

(**e**)

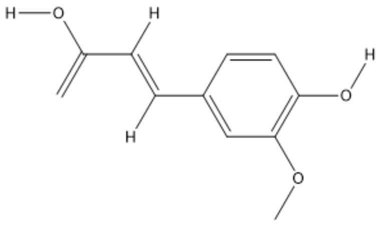

(**f**)

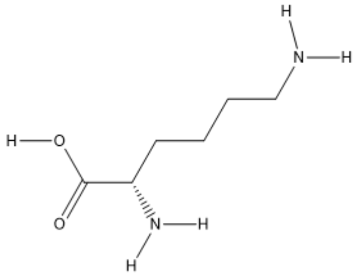

(**g**)

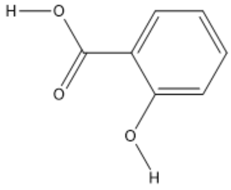

(**h**)

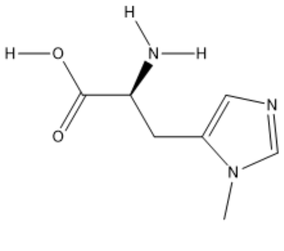

(**i**)

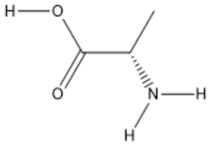

(**j**)

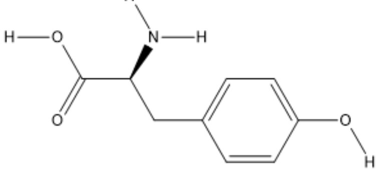

(**k**)

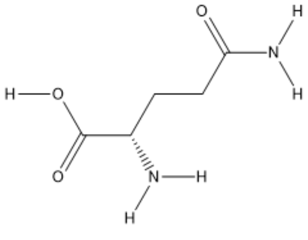

(**l**)

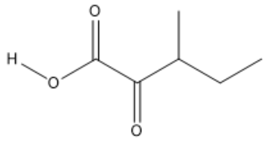

(**m**)

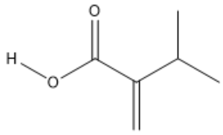

(**n**)

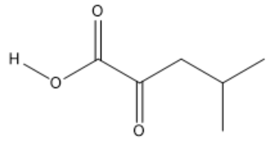

(**o**)

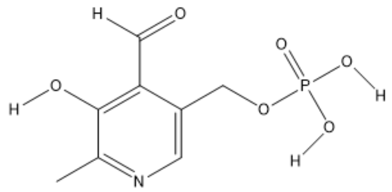

(**p**)

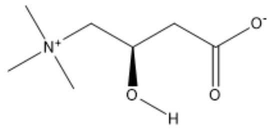

(**q**)

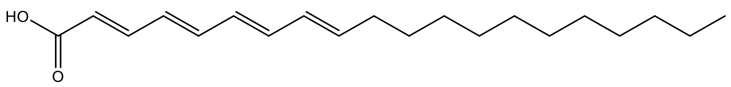

(**r**)

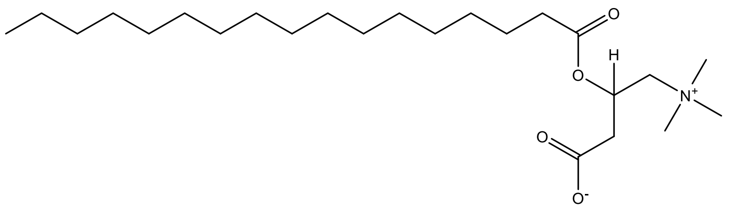

(**s**)

Figure S2. Amino Acids: (a) chemical structure of valine; (b) chemical structure isoleucine; (c) chemical structure of leucine; (d) chemical structure of phenylalanine; (e) chemical structure of D-glutamic acid; (f) chemical structure of ferulic acid; (g) chemical structure of lysine; (h) chemical structure of salicylic acid; (i) chemical structure of 3-methylhistidine; (j) chemical structure of alanine; (k) chemical structure of tyrosine; (l) chemical structure of glutamine; (m) chemical structure of α-keto-β-methylvalerate; (n) chemical structure of α-ketoisovalerate; (o) chemical structure of α-ketoisocaproate; (p) chemical structure of Pyridoxal 5′-phosphate; (q) chemical structure of L-Carnitine; (r) chemical structure of eicosatetraenoic acid; (s) chemical structure of acylcarnitines.

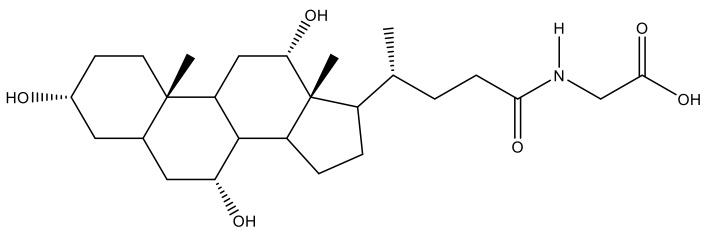
OH
HO
OH
H
N
O
O
OH

(**a**)

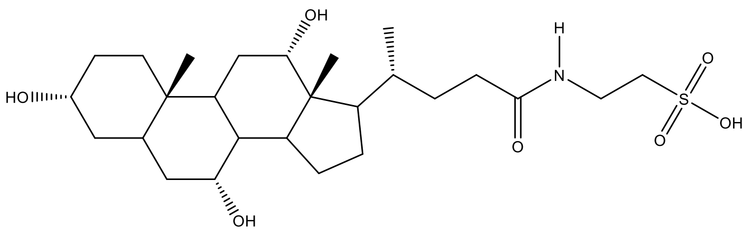
OH
HO
OH
H
N
O
S
O
O
OH

(**b**)

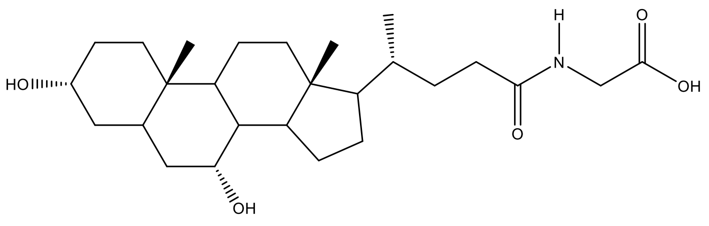
HO
OH
H
N
O
O
OH

(**c**)

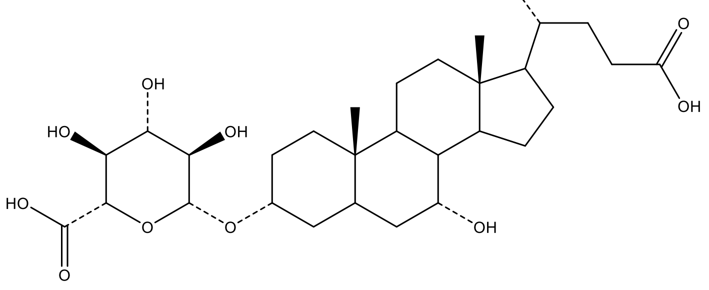
OH
HO
OH
HO
O
O
O
OH
O
OH

(**d**)

Figure S3. Steroid acids: (a) chemical structure of glycocholic; (b) chemical structure of taurocholic acid; (c) chemical structure of glycochenodeoxycholic acid; (d) chemical structure of chenodeoxycholic ac-id-3-β-d-glucuronide.
